# Evaluation of organ glucose metabolism by ^18^F-FDG accumulation with insulin loading in aged mice compared with young normal mice

**DOI:** 10.1038/s41598-021-86825-8

**Published:** 2021-04-01

**Authors:** Jingmin Zhao, Chengbo Tan, Ryota Imai, Naoyuki Ukon, Saki Shimoyama, Yuko Maejima, Yuji Omiya, Kazuhiro Takahashi, Hiroshi Ito, Guangxian Nan, Songji Zhao, Kenju Shimomura

**Affiliations:** 1 Department of Neurology, China-Japan Union Hospital of Jilin University, 126 XianTai Street, Changchun, 130031 Jilin China; 2 Department of Radiology and Nuclear Medicine, Fukushima Medical University, Fukushima, Japan; 3 Department of Neurosurgery, Shanghai East Hospital, Tongji University School of Medicine, Shanghai, China; 4 Advanced Clinical Research Center, Fukushima Global Medical Science Center, Fukushima Medical University, Fukushima, 960-1295 Japan; 5 Department of Bioregulation and Pharmacological Medicine, Fukushima Medical University, Fukushima, Japan; 6 Tsumura Kampo Research Laboratories, Kampo Research & Development Division, Tsumura & Co., Ibaraki, Japan; 7 Basic Medical College of Jilin University, Changchun, China

**Keywords:** Biomarkers, Molecular medicine

## Abstract

It is important to determine the functional changes of organs that occur as a result of aging, the understanding of which may lead to the maintenance of a healthy life. Glucose metabolism in healthy bodies is one of the potential markers used to evaluate the changes of organ function. Thus, information about normal organ glucose metabolism may help to understand the functional changes of organs. [^18^F]-Fluoro-2-deoxy-2-d-glucose (^18^F-FDG), a glucose analog, has been used to measure glucose metabolism in various fields, such as basic medical research and drug discovery. However, glucose metabolism changes in aged animals have not yet been fully clarified. The aim of this study is to evaluate changes in glucose metabolism in organs and brain regions by measuring ^18^F-FDG accumulation and ^18^F-FDG autoradiography with insulin loading in aged and young wild-type mice. In the untreated groups, the levels of ^18^F-FDG accumulation in the blood, plasma, muscle, lungs, spleen, pancreas, testes, stomach, small intestine, kidneys, liver, brain, and brain regions, namely, the cortex, striatum, thalamus, and hippocampus, were all significantly higher in the aged mice. The treated group showed lower ^18^F-FDG accumulation levels in the pancreas and kidneys, as well as in the cortex, striatum, thalamus, and hippocampus in the aged mice than the untreated groups, whereas higher ^18^F-FDG accumulation levels were observed in those in the young mice. These results demonstrate that insulin loading decreases effect on ^18^F-FDG accumulation levels in some organs of the aged mice. Therefore, aging can increase insulin resistance and lead to systemic glucose metabolism dysfunction.

## Introduction

With a globally aging population, the health issues caused by aging and age-related diseases have become inevitable challenges for all countries. Understanding the functional changes of organs that occur as a result of aging is essential to prevent these age-related diseases, including Alzheimer's disease (AD), Parkinson's disease (PD), dementia, stroke, peripheral neuropathy, macular degeneration, cataracts, senile deafness, diabetes mellitus, osteoporosis, osteoarthritis, atherosclerosis, prostatic hyperplasia, and even cancer^1^. In particular, the metabolism of glucose as an energy source has been regarded as a potential indicator for these disorders.

Under tight hormonal control by insulin, glucose homeostasis is maintained by a balance among glucose ingestion, utilization, and production^2^. As age advances, glucose homeostasis tends to gradually become disrupted, giving rise to type 2 diabetes (T2D) and cardiovascular diseases^3–5^. Insulin resistance is one of the major mechanisms underlying abnormal glucose tolerance^6^. Recognition of abnormal glucose metabolism in the elderly is important in implementing age-appropriate preventive and therapeutic strategies. It is widely accepted that aging is accompanied by an increase in insulin resistance^7,8^. This age-related insulin resistance has been variously attributed to several factors, including mitochondrial dysfunctions, reduced lean muscle mass and elevated adiposity, hormonal changes, increased oxidative stress, inflammation, and reduced physical activity^9–14^. However, these studies do not provide insights into organ-specific differences in insulin resistance.

The glucose analog [^18^F]-Fluoro-2-deoxy-2-d-glucose (^18^F-FDG), a molecular imaging probe, is widely used in nuclear medicine for evaluating tissue glucose utilization and glucose metabolism^15–17^. Although ^18^F-FDG accumulation has been investigated in various fields, such as basic medical research and drug discovery, changes in glucose metabolism evaluated using ^18^F-FDG in aged animals have not yet been fully clarified. Determining whether ^18^F-FDG distribution is associated with aging could provide insight into metabolic changes and help to prevent age-related diseases.

Therefore, in this study, we attempted to clarify the changes in glucose metabolism that occur with aging by comparing ^18^F-FDG accumulation levels after insulin loading in aged and young wild-type mice.

## Results

### Organ ^18^F-FDG accumulation experiment in control groups

The body weight and blood glucose level were determined in young and aged mice groups (Table 1).

**Table 1:** Body weight (g) and blood glucose concentration (mg/dl) in organ ^18^F-FDG accumulation study.

	Young group		Aged group	
	Control (n = 5)	Insulin (n = 5)	Control (n = 5)	Insulin (n = 4)
Body weight	22.0 ± 0.9	22.2 ± 1.2	30.04 ± 0.8^####^	31.9 ± 2.4^####^
Blood glucose	91.4 ± 9.9	24.0 ± 6.8****	95.0 ± 13.6	22.3 ± 3.9****

Data in parentheses are mean ± SD.

Control, control group; Insulin, insulin-loaded group.

****P < 0.0001 vs control value.

^#^^###^*P* < 0.0001 vs young groups in control and insulin-loaded groups.

The weight of organ level was determined in young and aged mice groups (Table 2). The levels of organ ^18^F-FDG accumulation were determined in young and aged mice groups (Table 3). The body weight of the aged mice was significantly higher than that of the young mice (Table 1). The blood glucose concentration was not significantly different between two groups (Table 1). The weights of the heart, lungs, spleen, pancreas, white adipose tissue, testes, stomach, small intestine, kidneys and liver were significantly higher in the aged mice than in the young mice (Table 2). Apart from the white adipose tissue, brown adipose tissue, large intestine, and heart, the levels of ^18^F-FDG uptake in the blood, plasma, muscle, lungs, spleen, pancreas, testes, stomach, small intestine, kidneys, liver and brain were all significantly higher in the aged mice than in the young mice (Table 3).

**Table 2:** Weights of organs in mice (g).

	Young groups		Aged groups	
	Control (n = 5)	Insulin (n = 5)	Control (n = 5)	Insulin (n = 4)
Muscle	0.128 ± 0.019	0.116 ± 0.019	0.136 ± 0.023	0.125 ± 0.036
Heart	0.088 ± 0.008	0.093 ± 0.007	0.132 ± 0.013***	0.129 ± 0.007**
Lung	0.115 ± 0.010	0.105 ± 0.009	0.160 ± 0.013***	0.155 ± 0.013**
Spleen	0.049 ± 0.004	0.047 ± 0.006	0.086 ± 0.018**	0.067 ± 0.013*
Pancreas	0.091 ± 0.023	0.099 ± 0.027	0.163 ± 0.018***	0.173 ± 0.016**
White adipose tissue (superior pole of epididymis)	0.070 ± 0.014	0.081 ± 0.014	0.141 ± 0.055*	0.185 ± 0.055**
Testis	0.086 ± 0.007	0.079 ± 0.011	0.097 ± 0.003*	0.096 ± 0.007*
Stomach	0.110 ± 0.008	0.119 ± 0.009	0.164 ± 0.011****	0.181 ± 0.011****
Small intestine	0.825 ± 0.042	0.891 ± 0.092	1.224 ± 0.021****	1.343 ± 0.126***
Large intestine	0.115 ± 0.010	0.111 ± 0.010	0.129 ± 0.012	0.138 ± 0.013**
Kidney	0.264 ± 0.005	0.277 ± 0.014	0.433 ± 0.022****	0.476 ± 0.086**
Liver	0.885 ± 0.029	0.895 ± 0.063	1.134 ± 0.073****	1.125 ± 0.240
Brown adipose tissue (between the shoulder blades)	0.053 ± 0.006	0.049 ± 0.011	0.057 ± 0.013	0.062 ± 0.013
Brain	0.331 ± 0.016	0.330 ± 0.014	0.345 ± 0.017	0.340 ± 0.023

Data in parentheses are mean ± SD.

Control, control group; Insulin, insulin-loaded group.

*P < 0.05, ** *P* < 0.01, ***P < 0.001, ****P < 0.0001 vs young groups in control and insulin-loaded groups.

**Table 3:** Organ ^18^F-FDG accumulation in mice (%ID/g/kg).

	Young groups		Aged groups	
	Control (n = 5)	Insulin (n = 5)	Control (n = 5)	Insulin (n = 4)
Blood	0.019 ± 0.002	0.010 ± 0.000****	0.033 ± 0.002	0.012 ± 0.002****
Blood plasma	0.015 ± 0.002	0.007 ± 0.001****	0.027 ± 0.003	0.010 ± 0.002****
Muscle	0.016 ± 0.003	0.052 ± 0.005****	0.028 ± 0.007	0.057 ± 0.019*
Heart	0.473 ± 0.253	1.538 ± 0.211****	0.479 ± 0.259	1.551 ± 0.406**
Lung	0.112 ± 0.013	0.102 ± 0.014	0.147 ± 0.011	0.108 ± 0.023*
Spleen	0.095 ± 0.011	0.064 ± 0.008**	0.141 ± 0.021	0.072 ± 0.012***
Pancreas	0.053 ± 0.005	0.077 ± 0.015*	0.072 ± 0.010	0.063 ± 0.004
White adipose tissue (superior pole of epididymis)	0.007 ± 0.002	0.031 ± 0.003****	0.010 ± 0.004	0.033 ± 0.008***
Testis	0.133 ± 0.011	0.024 ± 0.003****	0.185 ± 0.014	0.035 ± 0.005****
Stomach	0.077 ± 0.009	0.137 ± 0.010****	0.104 ± 0.018	0.195 ± 0.064*
Small intestine	0.105 ± 0.014	0.084 ± 0.014	0.147 ± 0.019	0.088 ± 0.020**
Large intestine	0.187 ± 0.012	0.153 ± 0.023*	0.202 ± 0.034	0.154 ± 0.027
Kidney	0.057 ± 0.006	0.133 ± 0.033***	0.084 ± 0.010	0.080 ± 0.046
Liver	0.032 ± 0.002	0.016 ± 0.002****	0.060 ± 0.010	0.025 ± 0.008***
Brown adipose tissue (between the shoulder blades)	0.050 ± 0.009	0.289 ± 0.065****	0.051 ± 0.012	0.252 ± 0.099**
Brain	0.370 ± 0.037	0.488 ± 0.036***	0.488 ± 0.059	0.551 ± 0.190

Data in parentheses are mean ± SD.

Control, control group; Insulin, insulin-loaded group.

*P < 0.05, **P < 0.01, ***P < 0.001, ****P < 0.0001 vs control value.

### Changes in ^18^F-FDG accumulation level in organs

In the young mice group, the levels of ^18^F-FDG uptake in the muscle, heart, pancreas, white adipose tissue, stomach, kidneys, brown adipose tissue, and brain significantly increased after insulin loading. In contrast, the levels of ^18^F-FDG uptake in the blood, plasma, spleen, testes, large intestine, and liver significantly decreased after insulin loading (Table 3). On the other hand, in the aged mice group, the levels of ^18^F-FDG uptake in the muscle, heart, white adipose tissue, stomach, and brown adipose tissue significantly increased after insulin loading. However, the levels of ^18^F-FDG uptake in blood, plasma, spleen, testes, and liver were significantly decreased after insulin loading (Table 3). Compared with the young mice group, the levels of ^18^F-FDG uptake in the pancreas, kidneys and brain did not exhibit observable changes after insulin loading in the aged mice group. In contrast, those in the lungs and small intestine significantly decreased after insulin loading (Table 3).

**Figure 1:**
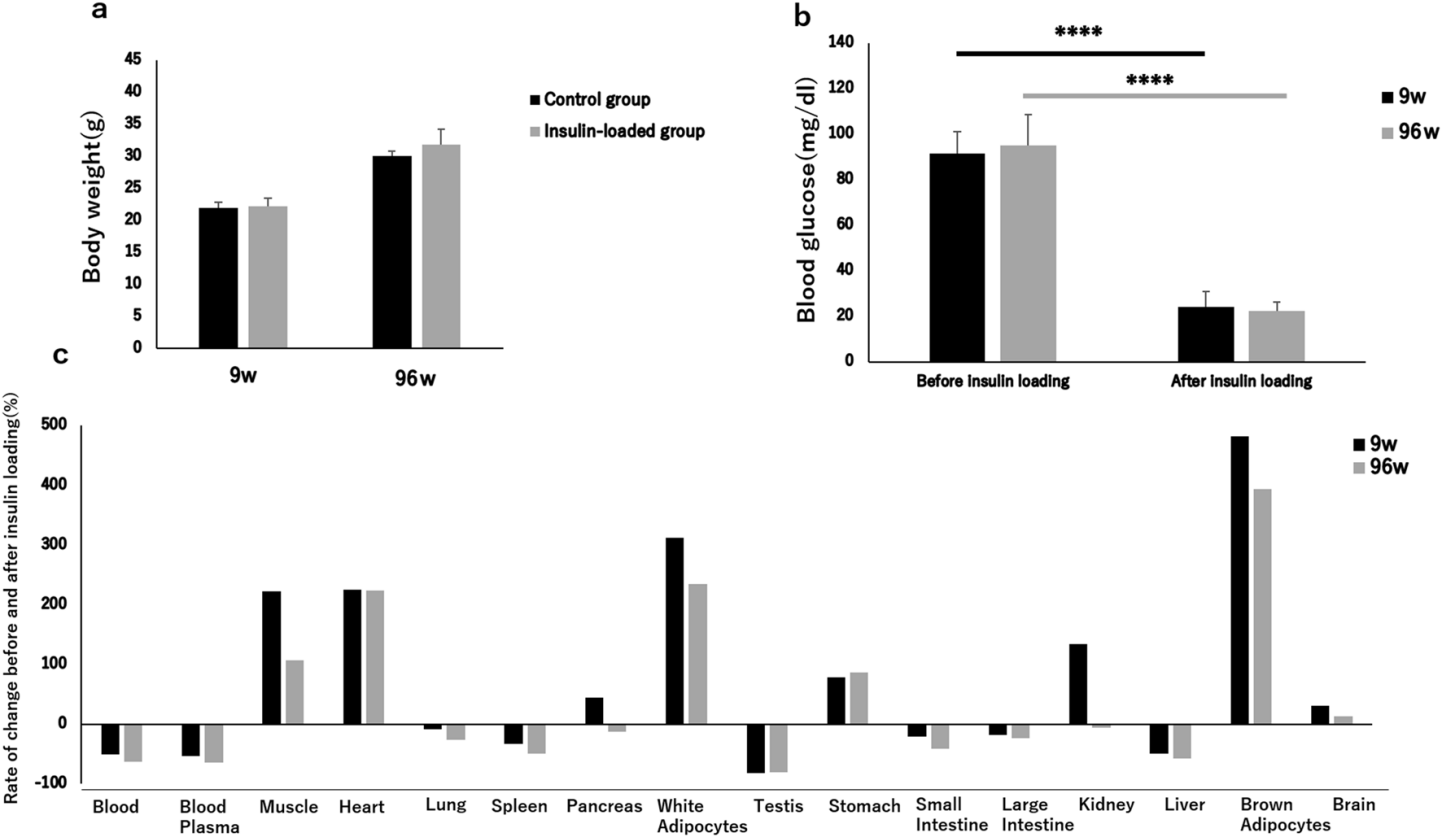
Body weight (**a**), blood glucose concentration (**b**), and rate of ^18^F-FDG uptake changes (**c**) in organs in young and aged control and insulin-loaded groups. ****P < 0.0001.

The body weight showed no significant difference between control and insulin-loaded groups in both young and aged mice (Table 1, Fig. 1a). When the blood glucose concentration decreased and displayed as “low” after insulin loading, we defined this blood glucose concentration as 20 mmol/dl which is the detection limit of blood glucose meter. After insulin loading, the blood glucose concentration significantly decreased in both young and aged mice groups (Table 1, Fig. 1b). The rate of ^18^F-FDG uptake level change in the control and insulin-loaded groups was assessed according to the following formula: ([mean level of ^18^F-FDG uptake in insulin-loaded group − mean level of ^18^F-FDG uptake in control group]/mean level of ^18^F-FDG uptake in control group) × 100%. In these mice, the rates of change in the muscle, heart, white adipose tissue, stomach, brown adipose tissue, and brain showed positive changes, whereas those in the blood, plasma, lungs, spleen, testes, small intestine, large intestine, and liver showed negative changes (Fig. 1c). Moreover, the rates of change in the pancreas and kidneys showed positive changes in the young mice but negative changes in the aged mice (Fig. 1c). Regarding these positive changes, the rate of change in the muscle in the young mice was higher than that in the aged mice (223.3% vs 107.9%), as well as brain (31.6%vs 13.0%), white adipose tissue (313.1% vs 235.6%) and brown adipose tissue (482.9% vs 394.6%) (Fig. 1c).

### Brain ^18^F-FDG autoradiographic experiment in control groups

**Figure 2:**
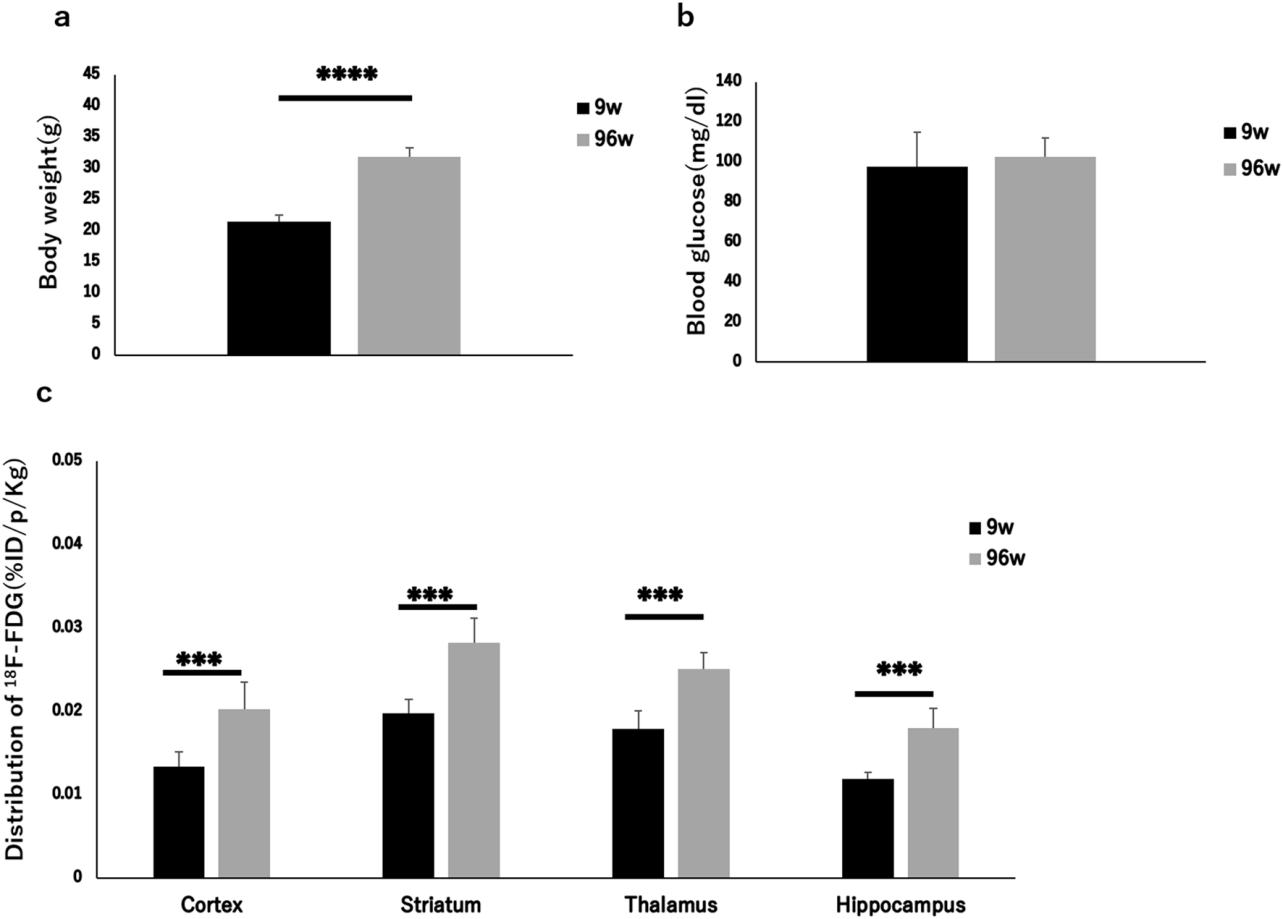
Body weight (**a**), blood glucose concentration (**b**), and ^18^F-FDG distribution (**c**) in brains in young and aged control groups. ***P < 0.001, ****P < 0.0001.

The body weight and blood glucose concentration were determined in the young and aged mice (Table 4). The levels of ^18^F-FDG uptake in brain regions were determined in the young and aged mice (Table 5). The body weight of the aged mice was significantly higher than that of the young mice (*P* < 0.0001) (Table 4, Fig. 2a). The blood glucose concentration was not significantly different between the two groups (Table 4, Fig. 2b). The levels of ^18^F-FDG accumulation in the cortex, striatum, thalamus, and hippocampus were all significantly higher in the aged mice than in the young mice (*P* < 0.001) (Table 5, Fig. 2c).

**Table 4:** Body weight (g) and blood glucose concentration (mg/dl) in brain ^18^F-FDG autoradiography study.

	Young group		Aged group	
	Control (n = 6)	Insulin (n = 6)	Control (n = 6)	Insulin (n = 6)
Body weight	21.4 ± 1.0	22.0 ± 0.7	31.8 ± 1.5^####^	29.7 ± 1.9^####^
Blood glucose	97.8 ± 17.2	21.5 ± 3.7****	102.5 ± 9.5	22.5 ± 6.1****

Data are shown in parentheses (mean ± SD).

Control, control group; Insulin, insulin-loaded group.

****P < 0.0001 vs control value.

^#^^###^*P* < 0.0001 vs young groups in control and insulin-loaded groups.

**Table 5:** ^18^F-FDG accumulation in brain regions in mice (%ID/p/kg).

	Young group		Aged group	
	Control (n = 6)	Insulin (n = 6)	Control (n = 6)	Insulin (n = 6)
Cortex	0.013 ± 0.002	0.017 ± 0.002*	0.020 ± 0.003	0.015 ± 0.004*
Striatum	0.020 ± 0.002	0.024 ± 0.002***	0.028 ± 0.003	0.026 ± 0.005
Thalamus	0.018 ± 0.002	0.020 ± 0.002	0.025 ± 0.002	0.021 ± 0.007
Hippocampus	0.012 ± 0.001	0.016 ± 0.001****	0.018 ± 0.002	0.015 ± 0.003

Data are shown in parentheses (mean ± SD).

Control, control group; Insulin, insulin-loaded group.

*P < 0.05, ***P < 0.001, ****P < 0.0001 vs control value.

### Changes in ^18^F-FDG accumulation level in brain regions

**Figure 3:**
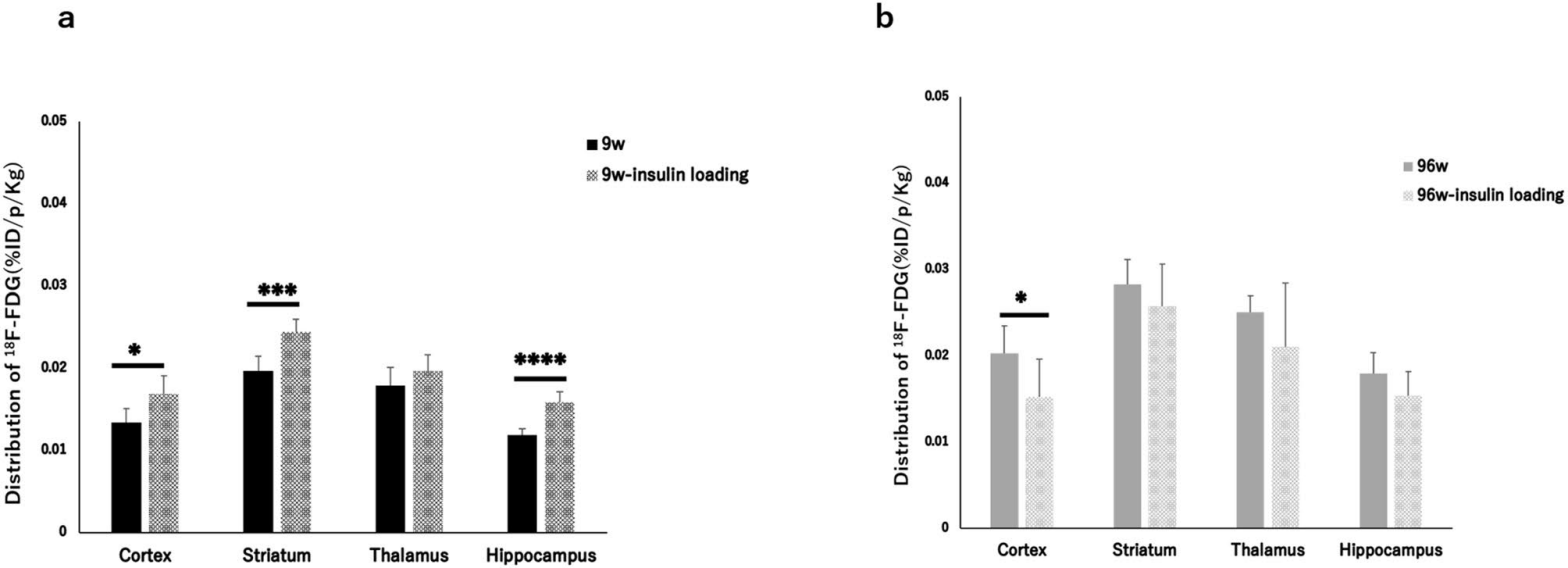
^18^F-FDG distribution in brains with and without insulin loading in young and aged groups. *P < 0.05, ***P < 0.001, ****P < 0.0001.

The levels of ^18^F-FDG accumulation in brain regions in the insulin-loaded young and aged mice were determined and compared with those in the control young and aged groups (Table 5). In the brain, the levels of ^18^F-FDG accumulation in the cortex, striatum, and hippocampus significantly increased after insulin loading in the young group (Table 5, Fig. 3a). Compared with the young group, the levels of ^18^F-FDG accumulation in the striatum, thalamus and hippocampus did not show observable changes after insulin loading in the aged group (Table 5, Fig. 3b). In contrast, the level of ^18^F-FDG accumulation in the cortex significantly decreased after insulin loading in the aged group (Table 5, Fig. 3b).

**Figure 4:**
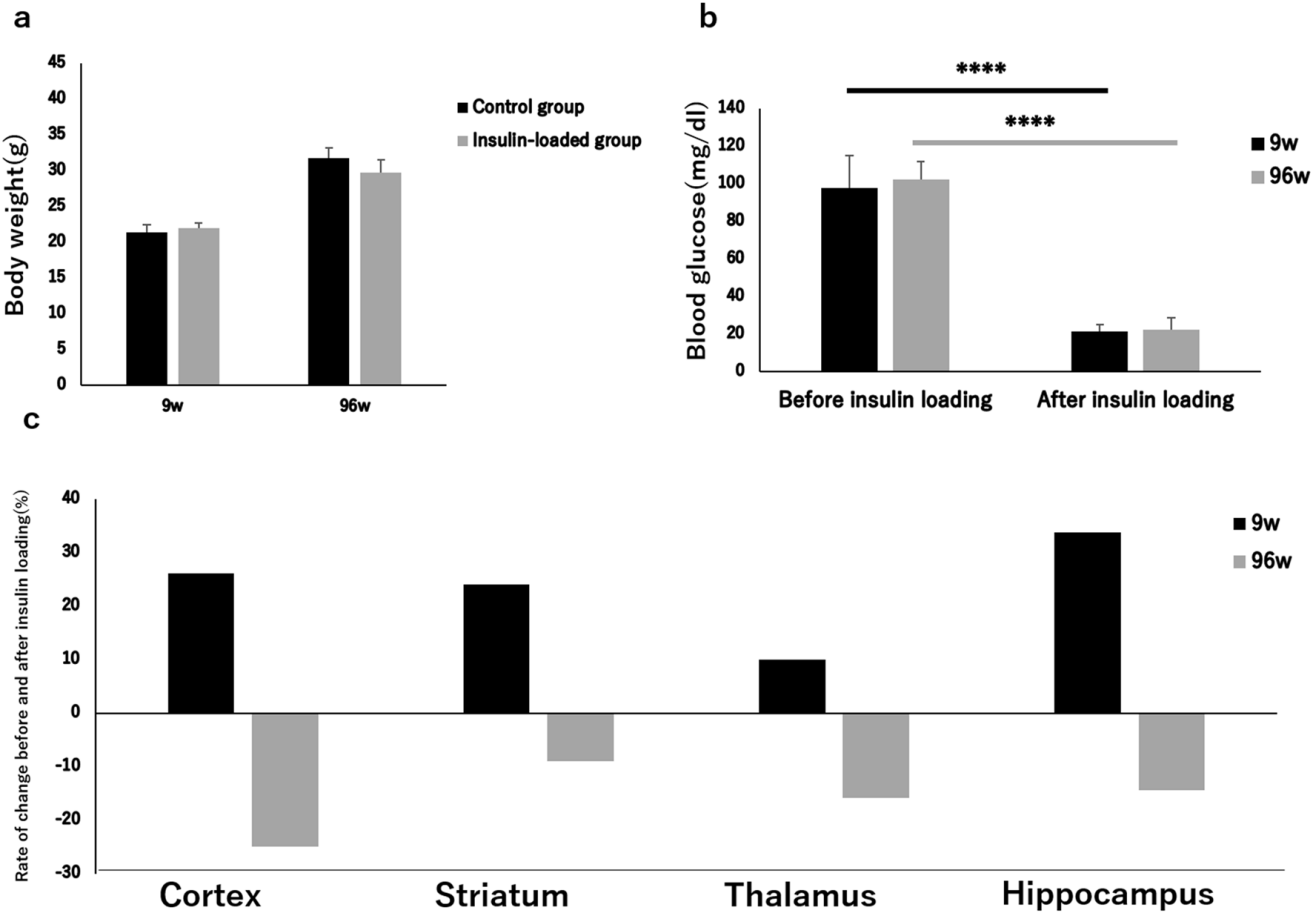
Body weight (**a**), blood glucose concentration (**b**), and rate of ^18^F-FDG accumulation changes (**c**) in brains in young and aged control and insulin-loaded groups. ****P < 0.0001.

The body weight showed no significant difference between control and insulin-loaded groups in both young and aged mice (Table 4, Fig. 4a). When the blood glucose concentration decreased and displayed as “low” after insulin loading, we defined this blood glucose concentration as 20 mmol/dl which is the detection limit of blood glucose meter. After insulin loading, the blood glucose concentration significantly decreased in both young and aged groups (Table 4, Fig. 4b). The rate of ^18^F-FDG accumulation level change in the control and insulin-loaded groups was assessed according to the following formula: ([mean level of ^18^F-FDG accumulation in insulin loaded group − mean level of ^18^F-FDG accumulation in control group]/mean level of ^18^F-FDG accumulation in control group) × 100%. The ^18^F-FDG accumulation level showed positive changes in the cortex, striatum, thalamus, and hippocampus in the young group, whereas negative changes were observed in those in aged group (Fig. 4c).

## Discussion

Alterations in glucose homeostasis are enhanced with age and can be linked to T2D, cardiocerebrovascular injury, and other age-related diseases^18^. To clarify the changes in glucose metabolism with aging, we examined and compared the body weight, blood glucose concentration, and ^18^F-FDG accumulation level in each organ between the aged and young mice. The age range of aged mice is defined as 18–24 months^19^. On the basis of his standard, we chose the 96-week-old male C57BL/6J mice as the aged group.

In this study, the body weight of the aged mice was markedly higher than that of the young mice. Previous studies have indicated that the progressive decline in insulin action with age can be attributed largely to gradual increases in the degree of relative obesity and the number of sites of fat deposition. In addition, insulin resistance is associated with sarcopenia and an accompanying relative increase in fat mass^20,21^. In a longitudinal study of over 4500 healthy individuals, Lindstrom and Tuomilehto^22^ and Salmon^23^ showed that the likelihood of patients aged 55–64 years developing drug-treated diabetes was roughly equivalent to patients with a BMI > 30. Since muscle is a crucial variable in determining the efficacy of glucose uptake, a decrease in muscle mass leads to a simultaneous decrease in glucose disposal rate^24,25^. Although the mechanisms underlying the link between insulin resistance and fat deposition have not yet been fully elucidated, it has been suggested that because of both an increased level of the β-adrenoceptor pathway and a reduced α2-adrenoceptor component level, visceral adipose tissue shows a high lipolytic response to catecholamine, exposing the liver to high free fatty acid concentrations, which in turn induces insulin resistance^25–28^. In this study, after insulin loading, the muscle ^18^F-FDG uptake rate of aged mice was significantly lower than that of young mice (107.9% vs 223.3%). The organ weight of the white adipose tissue was significantly higher in the aged mice than in the young mice. In aged mice, the increase in adipose tissue weight may lead to insulin resistance to decrease glucose uptake level in the muscle.

In all the insulin-loaded groups, the levels of ^18^F-FDG uptake in the muscle, heart, white adipose tissue, stomach, and brown adipose tissue were significantly higher than in all the control groups. Glucose transporter type 4 (Glut-4), which is an insulin-regulated glucose transporter, is primarily expressed in several tissues, including adipocytes, as well as the skeletal and cardiac muscles^29,30^. An excessively high blood insulin concentration leads to a series of signal cascades, including the autophosphorylation of the insulin cell surface receptor, the activation of receptor tyrosine kinase, the tyrosine phosphorylation of insulin receptor substrates 1 and 2, the activation of phosphatidylinositol 3-kinase, and the activation of protein kinase B and its downstream mediator AS160. These signal cascades eventually induce the translocation of a large quantity of Glut-4 from intracellular vesicles to the plasma membrane^31,32^. Thus, hyperinsulinemia markedly increases the level of glucose uptake in adipocytes and the skeletal and cardiac muscles.

In all the insulin-loaded groups, the levels of ^18^F-FDG uptake in the blood, plasma, spleen, testes, and liver were significantly lower than those in all the control groups. Previous studies ascribed the reduced ^18^F-FDG uptake in tumors and inflammatory lesions with insulin-induced hypoglycemia to the effects of insulin, which shifts ^18^F-FDG from the original area to insulin-sensitive organs^33,34^. This insulin effect may also explain the reduced ^18^F-FDG accumulation in insulin-insensitive organs with insulin-induced hypoglycemia.

In the control groups, excluding the heart, white adipose tissue, brown adipose tissue, and large intestine, the levels of ^18^F-FDG accumulation in the blood, plasma, muscle, lungs, spleen, pancreas, testes, stomach, small intestine, kidneys, liver, and brain were all significantly higher in the aged mice than that in the young mice. However, the blood glucose concentration was not significantly different between the young and aged control groups. Previous comparative studies have shown that C3H and CBA mice were among the most susceptible among all inbred mouse strains to hepatocarcinogenesis whereas the C57BL/6, A, BALB/c, and DBA/2 strains are relatively resistant, SWR/J mice were the most susceptible to lung tumor whereas the C57BR/cdJ, C57BL/6J, P/J, and SM/J strains were relatively resistant^35^. We observed no tumors in any organs of aged mice in this study. Hyperinsulinemia has been implicated in the progression of obesity, insulin resistance, and T2D. Elevated insulin levels can be a cause and consequence of obesity and insulin resistance^36–38^. Hyperinsulinemia, which is inevitably associated with insulin resistance, also appears to negatively affect kidney function via the induction of glomerular hyperfiltration and increase in vascular permeability^39^. In this study, the levels of ^18^F-FDG uptake in the kidneys in the young insulin-loaded group were significantly higher than those in the young control group. Compared with the young group, the levels of ^18^F-FDG uptake in kidneys in the aged group did not markedly change after insulin loading. Moreover, the levels of ^18^F-FDG uptake in the kidneys showed positive changes in the young mice, but negative changes were observed in the aged mice after insulin loading. As mentioned above, insulin-induced hypoglycemia shifts ^18^F-FDG from the original area to insulin-sensitive organs^33,34^. This insulin effect may also explain the reduced ^18^F-FDG uptake in the kidneys in the aged mice, because the decreased insulin sensitivity of the kidneys leads to the shift of ^18^F-FDG to insulin-sensitive organs. These findings suggest that there were obstacles in glucose metabolism in the kidneys in aged mice. On the other hand, this insulin resistance in old mice negatively affects kidney function, which leads to the excretion disorders of ^18^F-FDG, and subsequently to higher levels of ^18^F-FDG accumulation in organs of elderly mice. Kidney dysfunction is a major cause of morbidity and mortality, whose prevalence is rising worldwide mainly because of the aging of populations^40^. However, the epidemics of abnormalities associated with insulin resistance^41^ might play a role in the increase in the prevalence of kidney dysfunction^42^. Insulin receptor substrate 1 plays a key role in insulin signaling and action in several organs including the kidneys^43^. Among the genes involved in the insulin signaling pathway is the gene encoding transmembrane glycoprotein ectonucleotide pyrophosphatase phosphodiesterase 1, which binds to and inhibits the insulin receptor and subsequent downstream insulin signaling and action in both cultured cells and animal models^44^. We consider that aging could induce insulin resistance through abnormal-insulin-signaling-related genes, and ultimately lead to glucose intolerance and kidney dysfunction. On the other hand, although there is convincing evidence that the kidney is an insulin-regulating organ, in which insulin regulates various functions, similar to traditional target organs, it is unclear whether the kidney is affected by insulin resistance in whole or in part^45^. The insulin receptor exists in two isoforms A and B, which are formed due to exclusion (isoform A) or inclusion (isoform B) of exon 11 of the insulin receptor gene. Insulin receptor-A is ubiquitously expressed, whereas insulin receptor-B is expressed largely in the classically insulin-sensitive tissues of liver, skeletal muscle and adipose tissue. Interestingly, insulin receptor-B is also expressed abundantly in the kidney^46^. According to a literature review, insulin receptor-A may increase with aging, thereby contributing to insulin resistance, insulin receptor-B downregulation may contribute to insulin resistance in aging^47^. According to the above literature, we consider that aging could down-regulate insulin receptor B and up-regulate insulin receptor A, leading to a decrease in insulin sensitivity in the kidneys.

In addition, after insulin loading, the brain glucose uptake rate in old mice was significantly lower than that in young mice (13.0% vs 31.6%). To more specifically evaluate the changes in glucose metabolism in the brain, we carried out ^18^F-FDG autoradiography to evaluate the ^18^F-FDG accumulation in brain regions after insulin loading in aged and young wild-type mice. We found that the levels of ^18^F-FDG accumulation in the cortex, striatum, thalamus, and hippocampus were all significantly higher in the aged mice than in the young mice. However, the blood glucose concentration was not significantly different between the young and aged groups. This is consistent with the finding that the levels of ^18^F-FDG uptake in the brain were significantly higher in the aged mice that in the young mice in the study on the ^18^F-FDG distribution in organs. In the young mice group, the levels of ^18^F-FDG accumulation in the cortex, striatum, and hippocampus significantly increased after insulin loading. Compared with the young group, the levels of ^18^F-FDG accumulation in the striatum, thalamus and hippocampus in the aged group did not markedly change after insulin loading. In contrast, the level of ^18^F-FDG accumulation in the cortex significantly decreased after insulin loading in the aged group. Aging is associated with reductions in the levels of both insulin and its receptor in the brain, which may even cause the brain to be in the state of insulin resistance^48–51^. Cholerton et al*.*^49^ indicated that chronic high levels of insulin and insulin resistance may exert a negative effect on several body systems, including the central nervous system, for some time prior to the onset of diabetes. There is increasing support to the idea that such early insulin abnormalities may be associated with the initiation of the cascade of the AD pathology in some individuals, years or even decades before the first clinical dementia symptoms are manifest^49^. Bingham et al*.*^16^ demonstrated an enhanced cerebral glucose metabolism that was particularly pronounced in the cortex following the administration of a low dose of insulin. The basis for brain-region-specific insulin effects on glucose metabolism may be attributable to the distribution of GLUTs^52,53^. Insulin-sensitive GLUTs 4 and 8 are selectively distributed in the brain, and insulin increases the levels of brain GLUT 4 expression and translocation^54^. In this study, ^18^F-FDG accumulation showed positive changes in the cortex, striatum, thalamus, and hippocampus in the young mice, whereas negative changes were observed in those in the aged mice after insulin loading. These indicate that the insulin sensitivity of these brain regions might gradually decrease with age and lead to age-related brain diseases such as AD and PD. The characteristics of glucose metabolism in the localized brain regions of these aged mice can be used to develop therapeutic models for age-related brain diseases.

The limitation of our study was the lack of insulin sensitivity assays, such as the intraperitoneal glucose tolerance test (IPGTT), intraperitoneal insulin tolerance test (IPITT), and plasma insulin measurements.

## Conclusions

In summary, we demonstrated that aging can induce insulin resistance and lead to dysfunction of systemic glucose metabolism. Insulin resistance could affect glucose metabolism and eventually cause age-related diseases. On the basis of findings, ameliorating this dysfunction may be a good preventive and therapeutic strategy for age-related diseases.

## Methods

### Preparation of animal models

The entire experimental protocols were approved by the Laboratory Animal Care and Use Committee of Fukushima Medical University (approval number 30021) and performed in accordance with the Guidelines for Animal Experiments at Fukushima Medical University. The study was also carried out in compliance with the ARRIVE guidelines. Eight-week-old and 96-week-old male C57BL/6J mice were purchased from Charles River Laboratories Japan, Inc. (Yokohama, Japan) and maintained in a specific-pathogen-free animal experiment facility. The room temperature was maintained between 23 and 25 °C, and the relative humidity was maintained between 45 and 55%. The institutional laboratory housing provided a 12-h light/dark cycle and met all the criteria of the Association for Assessment and Accreditation of Laboratory Animal Care (AAALAC) International (http://www.aaalac.org/)^55^. All animals were fasted overnight and then divided into four subgroups: young control group, aged control group, young insulin-loaded group, and aged insulin-loaded group.

### Organ ^18^F-FDG accumulation study

Eight-week-old and 96-week-old male mice (n = 5, each group) were assigned to the control and insulin-loaded groups. Those in the insulin-loaded groups were intraperitoneally injected with human insulin (2 U/kg body weight, Eli Lilly & Co., Kobe) 30 min prior to ^18^F-FDG injection. We excluded one of the 96-week-old mice in the insulin-loaded group because the blood glucose level did not decrease after insulin loading. Each animal was initially anesthetized with 4% isoflurane in air and maintained via spontaneous ventilation with 2% isoflurane in air. ^18^F-FDG (11.5 MBq/0.1 ml) was injected into the tail vein. Ninety minutes later, the animals were sacrificed and their organs were excised. The organs (muscle, heart, lungs, spleen, pancreas, white adipose tissue (superior pole of epididymis), testes, stomach, small intestine, large intestine, kidneys, liver, brown adipose tissue (between the shoulder blades), and brain) and blood samples (blood and plasma) were weighed, and their radioactivity was determined with a gamma counter (WIZARD^2^ 2480; PerkinElmer, USA). After decay correction, the percentage of injected dose per gram of tissue was obtained and normalized to the animal weight [%ID/g tissue/kg body weight (%ID/g/kg)]. Blood samples for glucose concentration measurement were obtained from the control group and insulin-loaded groups.

### Brain ^18^F-FDG autoradiography study

**Figure 5:**
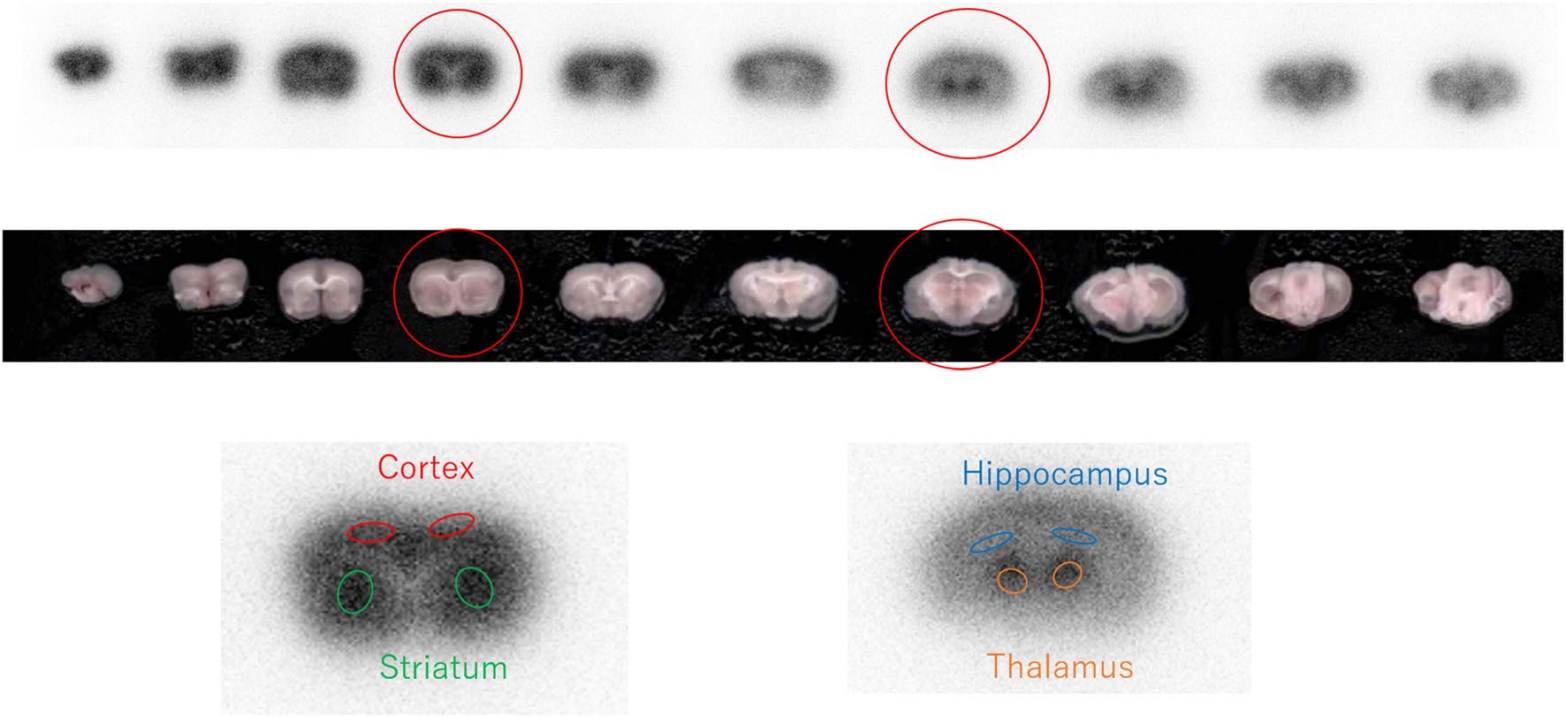
Brain ^18^F-FDG autoradiography image. ROIs were placed on ^18^F-FDG ARG image to cover the cortex, striatum, hippocampus, and thalamus on the left and right hemispheres. The cortex is encircled in red, the striatum in green, the hippocampus in blue, and the thalamus in orange.

Eight-week-old and 96-week-old male mice (n = 6, each group) were assigned to the control and insulin-loaded groups. Those in the insulin-loaded groups received an intraperitoneal injection of human insulin (2 U/kg body weight, Eli Lilly & Co., Kobe) 30 min prior to ^18^F-FDG injection. Each animal was initially anesthetized with 4% isoflurane in air and maintained via spontaneous ventilation with 2% isoflurane in air. ^18^F-FDG (11.5 MBq/0.1 ml) was injected into the tail vein. Ninety minutes later, the animals were sacrificed, then brains were rapidly removed, placed in Brain Matrix (Stoelting Co, USA) and cut into coronal slices (2 mm/slice) to obtain 8–9 coronal slices that were exposed to a phosphor imaging plate (Fuji Imaging Plate BAS-SR 2025 for ^18^F; Fuji Photo Film Co., Ltd., Tokyo, Japan) with a set of calibrated standards^56^. This autoradiographic exposure was performed overnight to detect the distribution of ^18^F-FDG. Autoradiography images were analyzed using a computerized imaging analysis system (raytest, CR35, Version 2.1.0, Straubenhardt, Germany) with the image analysis software AIDA (Version 5.1 SP2, Straubenhardt, Germany). To determine brain radioactivity concentration, the cortex, striatum, thalamus and hippocampus were defined using Aida Image Analyzer software. The regions of interests (ROIs), namely, the cortex, striatum, thalamus, and hippocampus in the left and right hemispheres in all mice were marked on the same anatomical plane with reference to the corresponding brain coronal slices (Fig. 5). The radioactivity in each ROI was determined per unit area, the percentage of injected dose per pixel of the cortex, striatum, thalamus, and hippocampus was obtained and normalized to the animal weight [%ID/pixel/kg body weight (%ID/p/kg)]. Finally, the average of the left and right values around each of the four regions was obtained. Blood samples for glucose concentration measurement were obtained from the control and insulin-loaded mice groups.

### Statistical analyses

All data are expressed as mean ± standard deviation. Statistical analyses were performed using the unpaired Student’s *t* test to evaluate the significance of differences between the young and aged mice in body weight, organ weights, blood glucose concentration, and ^18^F-FDG distribution, as well as between the control group and insulin-loaded group both in the young and aged mice groups. Significance was assumed at *P* < 0.05.

## Data Availability

The data generated and/or analyzed in this study are available from the corresponding author on reasonable request.
